# Cholangioscopy findings of biliary candidiasis post-liver transplantation

**DOI:** 10.1055/a-2261-7977

**Published:** 2024-02-22

**Authors:** Da Yeon Ryoo, Sylvester M. Black, Ashley Limkemann, Shaoli Sun, Nicole Gray, Samuel Han

**Affiliations:** 1 Department of Internal Medicine, The Ohio State University Wexner Medical Center, Columbus, United States; 2 Division of Transplantation Surgery, The Ohio State University Wexner Medical Center, Columbus, United States; 3 Department of Pathology, The Ohio State University Wexner Medical Center, Columbus, United States; 4 Division of Gastroenterology, Hepatology, and Nutrition, The Ohio State University Wexner Medical Center, Columbus, United States

Solid organ transplant patients are often subject to immunosuppressive agents, which predispose them to opportunistic infections. Of those infectious agents, fungal infections such as *Candida* are associated with the highest mortality rate 1 .

A 51-year-old man with a history of cirrhosis after orthotopic liver transplantation, with postoperative course complicated by bile leak that was treated with placement of a covered metal stent, was found on magnetic resonance imaging to have filling defects in the intrahepatic ducts suggestive of either debris or fungal balls ( Fig. 1 ). As the patient had persistent fungemia with *Candida glabrata* despite treatment, he underwent endoscopic retrograde cholangiopancreatography (ERCP) for further evaluation.

**Fig. 1:**
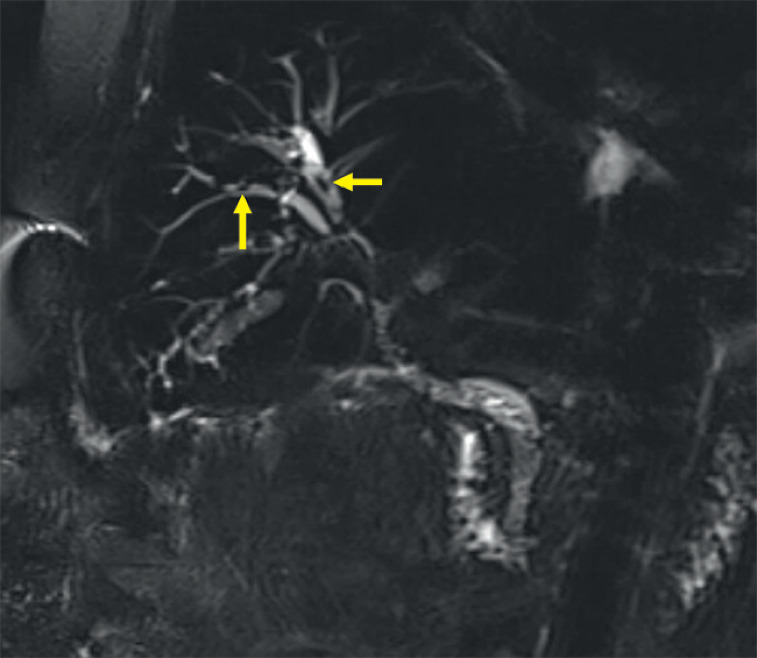
Magnetic resonance imaging revealing filling defects (arrows) within the intrahepatic ducts suggestive of debris or fungal balls.

Cholangioscopy ( Video 1 ) during ERCP revealed diffuse irregularities at the hepatic duct confluence, consisting of large amounts of brown fibrous tissue ( Fig. 2 ). Additionally, black streaks were scattered throughout the left hepatic ducts ( Fig. 3 ). Cholangioscopy-guided biopsies and brush cytology were obtained, as well as cultures from the biopsies and aspirated biliary fluid. Then, 500 mL of amphotericin B (50 mg/500 mL) were directly instilled into the intrahepatic ducts via the cholangioscope over 30 minutes.

Cholangioscopy findings of biliary candidiasis.Video 1

**Fig. 2:**
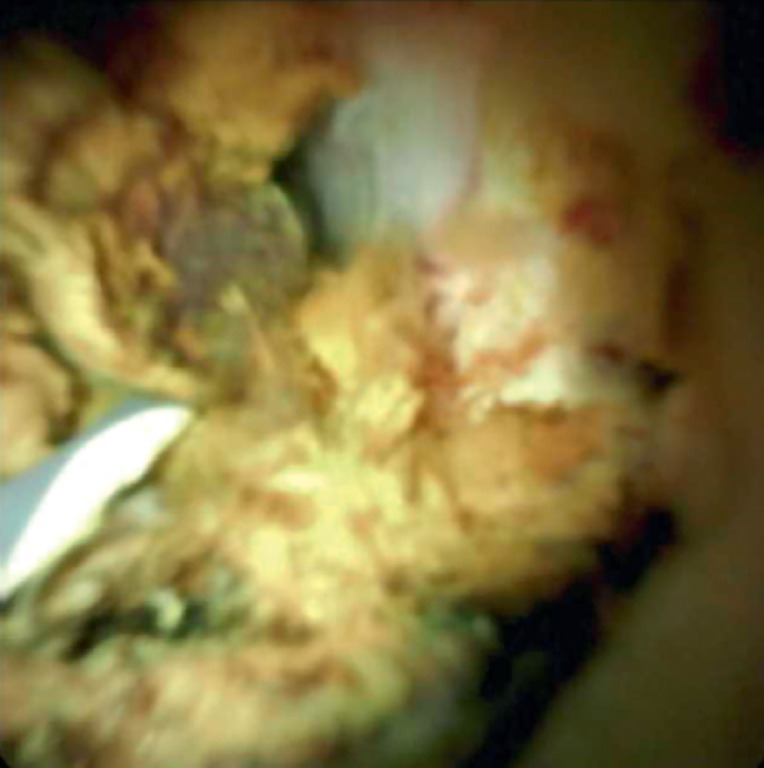
Brown strands of fibrous tissue found at the confluence, representative of fungal overgrowth.

**Fig. 3:**
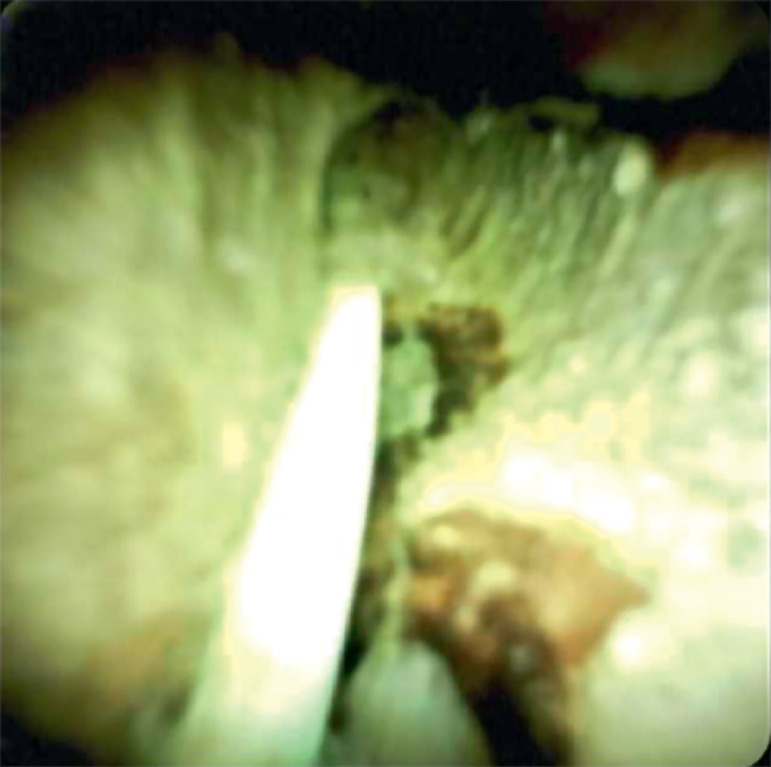
Streaks of black mucosa in the intrahepatic ducts, suggestive of ischemic injury.

Brush cytology and intraductal biopsies revealed fungal organisms morphologically consistent with *Candida* ( Fig. 4 ), in addition to ulcerated mucosa with fungal cultures growing *Candida glabrata* . As a salvage treatment, we repeated ERCP 1 week later with placement of a nasobiliary drain into the right hepatic duct. The patient received intrabiliary irrigation of amphotericin 12.5 mg three times daily, in addition to parenteral liposomal amphotericin and oral flucytosine, which resulted in resolution of the fungemia.

**Fig. 4:**
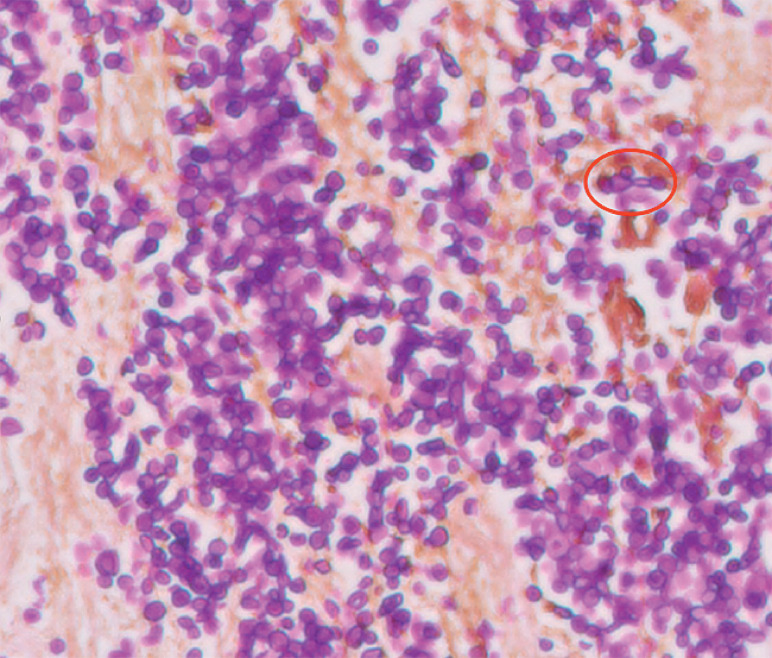
Yeast forms with pseudohyphae (arrow) visible on hepatic duct biopsies with periodic acid-Schiff stain with diastase.

Repeat ERCP after 2 weeks of this regimen showed significantly improved fibrous-like material in the right hepatic duct ( Fig. 5 ), with mild improvement in the left hepatic duct. Owing to persistent fungemia and bacteremia, however, the patient required repeat liver transplantation 3 months later.

**Fig. 5:**
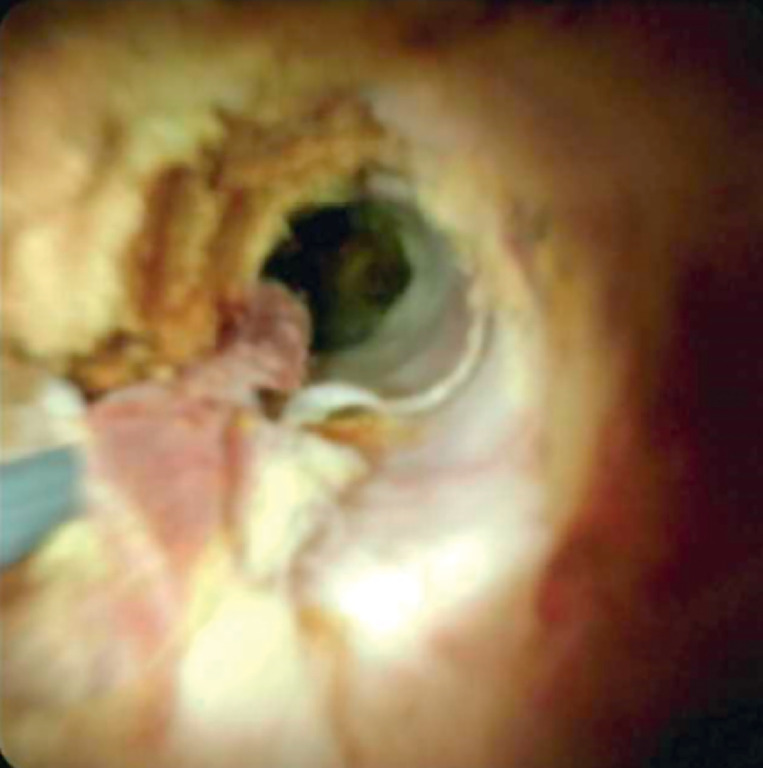
Improvement in fungal overgrowth found within the right hepatic duct on repeat cholangioscopy.

Endoscopy_UCTN_Code_TTT_1AR_2AK

## Notes

Endoscopy E-Videos https://eref.thieme.de/e-videos
*E-Videos* is an open access online section of the journal *Endoscopy* , reporting on interesting cases and new techniques in gastroenterological endoscopy. All papers include a high-quality video and are published with a Creative Commons CC-BY license. Endoscopy E-Videos qualify for HINARI discounts and waivers and eligibility is automatically checked during the submission process. We grant 100% waivers to articles whose corresponding authors are based in Group A countries and 50% waivers to those who are based in Group B countries as classified by Research4Life (see: https://www.research4life.org/access/eligibility/ ). This section has its own submission website at https://mc.manuscriptcentral.com/e-videos .
